# *Staphylococcus aureus* Small Colony Variants (SCVs): News From a Chronic Prosthetic Joint Infection

**DOI:** 10.3389/fcimb.2019.00363

**Published:** 2019-10-22

**Authors:** Guilherme Loss, Patricia Martins Simões, Florent Valour, Marina Farrel Cortês, Luiz Gonzaga, Marine Bergot, Sophie Trouillet-Assant, Jêrome Josse, Alan Diot, Emiliano Ricci, Ana Tereza Vasconcelos, Frédéric Laurent

**Affiliations:** ^1^Laboratório Nacional de Computação Científica, Rio de Janeiro, Brazil; ^2^National Reference Center for Staphylococci - Hospices Civils de Lyon, IAI-Department of Clinical Microbiology, Northern Hospital Group, Lyon, France; ^3^Centre International de Recherche en Infectiologie (CIRI), Lyon, France; ^4^Hospices Civils de Lyon, Infectious Diseases Department, Northern Hospital Group, Lyon, France; ^5^Institute of Microbiology Professor Paulo de Góes, Federal University of Rio de Janeiro, Rio de Janeiro, Brazil; ^6^Hospices Civils de Lyon, Joint Research Unit HCL-BioMerieux, Centre Hospitalier Lyon Sud, Pierre-Benite, France

**Keywords:** *Staphylococcus aureus*, small colony variant, prosthetic joint infection, chronic infection, rifampicin resistance

## Abstract

Small colony variants (SCV) of *Staphylococcus aureus* have been reported as implicated in chronic infections. Here, we investigated the genomic and transcriptomic changes involved in the evolution from a wild-type to a SCV from in a patient with prosthetic joint infection relapse. The SCV presented a stable phenotype with no classical auxotrophy and the emergence of rifampicin resistance. Whole Genome Sequencing (WGS) analysis showed only the loss of a 42.5 kb phage and 3 deletions, among which one targeting the *rpoB* gene, known to be the target of rifampicin and to be associated to SCV formation in the context of a constitutively active stringent response. Transcriptomic analysis highlighted a specific signature in the SCV strain including a complex, multi-level strategy of survival and adaptation to chronicity within the host including a protection from the inflammatory response, an evasion of the immune response, a constitutively activated stringent response and a scavenging of iron sources.

## Introduction

*Staphylococcus aureus* is among the most important human pathogens, associated with a wide variety of manifestations, ranging from benign superficial skin infections to life-threatening conditions, such as endocarditis, necrotizing pneumonia, and sepsis (Tong et al., 2015). Within the plethora of more severe conditions caused by *S. aureus*, bone and joint infections (BJIs) are caused by the invasion and progressive destruction of bone tissues and cartilages, mostly targeting long bones and native or prosthetic joints. Although BJI-related mortality is low, the development of these infections is burdened by a high morbidity with poor long-term functional outcome.

Despite the combination of an adequate surgical management with a prolonged course of antimicrobial chemotherapy, *S. aureus* BJIs are associated with a high propensity of chronicization and relapse (Osmon et al., 2013; Tande et al., 2014). Indeed, many clinical observational studies have demonstrated that *S. aureus* BJIs can persist asymptomatically before relapse occurs months or years after the initial episode, even in immunocompetent hosts (Greer and Rosenberg, 1993; Bosse et al., 2005; Stevens et al., 2007).

This capacity of *S. aureus* to persist and remain clinically quite indolent (Rogers et al., 2009) relies on alternative lifestyles adopted by the bacteria to protect itself from an inhospitable environment (Proctor et al., 2014). Among these lifestyles, the Small Colony Variants (SCV) phenotype can potentially be responsible for the chronicization of the infection (Garzoni and Kelley, 2009; Tande et al., 2014; Kahl et al., 2016), offering a better resistance to the challenge posed by the host defenses (Abel et al., 2011; Ou et al., 2016) and to antibiotic therapies (von Eiff et al., 1998; Massey et al., 2001; Massey and Peacock, 2002).

These SCVs variants constitute a naturally *quasi*-dormant, slow-growing subpopulation of bacteria. They harbor distinctive phenotypic and pathogenic traits such as formation of smaller colonies on agar plates, a quiescent metabolism, reduced haemolytic and coagulase activities, decreased carbohydrate utilization, low virulence potential, and elevated antibiotic resistance (Proctor et al., 2006; Johns et al., 2015). Recent work has reported that external environmental stress factors (reactive oxygen species, low pH, cationic peptides, and limited nutrition) can trigger the emergence of SCVs (Tuchscherr et al., 2010, 2011; Bui et al., 2015).

Environmental stressors induce the emergence of phenotypically distinct types of SCVs. Most of them represent a transient state that can, under favorable conditions, revert to a wild-type (WT) or a different phenotype (distinct from both the progenitor and the SCV), thus leading to the relapse that can be clinically observed. Others are due to permanent genetic changes, and are consequently stable, irreversible SCVs (von Eiff et al., 1998; Massey et al., 2001; Proctor et al., 2006; Tuchscherr et al., 2011; Onyango et al., 2013).

While phenotypic reversion occurs rapidly and is thought to circumvent any lasting fitness cost, permanent genetic alteration seems to depend largely on the nature of the environmental stress. Nevertheless, numbers of genetic changes are prevalent in SCV and related to auxotrophies, such as hemin, menadione, thiamine, thymidine, and/or unsaturated fatty acids (Proctor et al., 1995; Kohler et al., 2003; Seggewiss et al., 2006; Tuchscherr et al., 2011). More recently, these genetic changes have been associated with the persistent active stringent response coupled to *relA* and *rpoB* mutations (Gao et al., 2010). Novel mechanisms for the development of persistent organisms and SCVs have been reported in the last years and include changes in RNA processing, stringent response, toxin-antitoxin systems, ribosome protein L6 (RplF), or cold shock protein B (CspB) (for a review see: Proctor et al., 2014).

The detection and identification of SCVs in routine laboratories and their accurate studies in research laboratories are challenges not overcome yet. Indeed, the majority of studies conducted on SCVs use laboratory-generated mutant *S. aureus* strains rather than naturally derived SCVs. Recently Bui et al. published the first genomic comparison and transcriptomic analysis of a clinical “wild type” *S. aureus* isolate with its corresponding stable SCV. The latter was induced experimentally by a prolonged exposure of the parental form to a simulated inflammatory environment (methylglyoxal) and a steady-state growth conditions with low nutrients (Bui and Kidd, 2015). However, as for many previous works, the main drawback of this work is the lack of comparison between clinical acute and relapse/chronic SCV forms isolated from the same patient. Moreover, recent publications have revealed a number of mutations that result in SCVs, but most of these have not been studied in clinical isolates.

Here, we provide the first detailed description of the genetic and transcriptomic changes involved in the evolution of a natural wild type isolate into a stable SCV isolate in the clinical context of a relapsing prosthetic joint infection (PJI).

## Results and Discussion

### Isolation of *S. aureus* From Acute and Relapse Episodes of a Prosthetic Joint Infection: Clinical History, Antibiogram, and First Characterization

**Figure 1:**
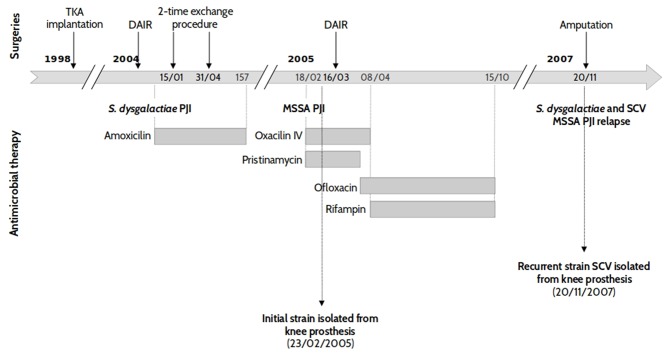
Patient history case and chronology of isolate collection. Two strains of *S. aureus* (SA) sensitive to methicillin (MSSA) were isolated from an 83 year-old patient with left knee prosthetic joint infection. The first isolate (strain ST20130944) was collected during the first, acute SA infection, while the second was obtained 2 years and 9 months later during a recurrent episode.

Two *S. aureus* clinical isolates were obtained from a patient who benefited from the implantation of a left total knee arthroplasty (TKA), see Figure 1. Six years after the implantation, the patient presented an acute haematogenous *Streptococcus dysgalactiae* PJI which, after an attempt for a conservative treatment, demonstrated signs of a persistent infection. TKA re-implantation occurred in March 2004 with an uneventful immediate postoperative course. Ten months later, the patient was admitted to the emergency department for acute fever associated with a painful and swollen left knee. All preoperative bacteriological samples yielded growth of a multi-susceptible *Staphylococcus aureus* (WT, isolate ST20130944). Treatment was interrupted in October 2005 after a 7 month course of antimicrobial therapy. However, the patient developed PJI relapse in June 2006, with no general symptoms. All the bacteriological samples made at the time of the surgery were positive to both *S. dysgalactiae* and *S. aureus*. The latter demonstrated the characteristics of a stable small colony variant (SCV, isolate ST20130945), as shown in Figure 1.

The *S. aureus* isolates from both the initial and relapse episodes were isolated from the same site of infections (knee prosthesis) and were, initially, characterized by DNA microarray and Protein A gene (*spa)* typing. Both strains presented the same DNA microarray profile with an assignment to CC30-MSSA [PVL-] and *spa* typing t12. These results suggest that the same *S. aureus* strain was responsible for both PJI episodes which were separated by 2 years and 9 months. Routine susceptibility tests were also performed to check if novel resistance phenotypes had emerged. Both the initial WT-strain (ST20130944) and relapse-SCV (ST20130945) isolates showed resistance to penicillin and tetracycline. The relapse SCV strain presented an additional resistance to rifampin.

### Exploration of the SCV Phenotype

**Figure 2:**
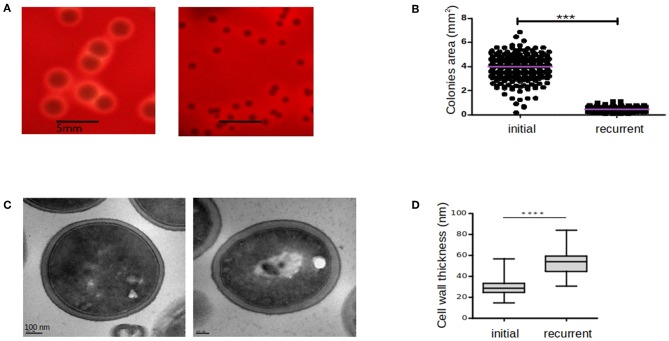
Comparison of colony sizes, hemolytic activities, and cell wall thickness between initial and recurrent infections. The initial-WT (ST20130944) and recurrent-SCV (ST20130945) strains were cultured on blood agar plates and hemolytic activity **(A)** and colony areas **(B)** were assessed after 48 h incubation at 37°C. Cell-wall thickness of ST20130944 (left hand-side) and ST20130945 (right hand-side) strain photographed by TEM **(C)** and comparison of cell-wall thickness of initial and recurrent strains **(D)**. Colony areas (mm^2^) were measured among three independent experiments and compared using the non-parametric exact Wilcoxon-Mann-Whitney test with alpha < 0.05. Thickness measurements (mm) were taken twice per cell in 30 cells per condition and compared using the non-parametric exact Wilcoxon-Mann-Whitney test with alpha < 0.05. Horizontal bars denote the 1st, 2nd (median), and 3rd quartiles. ***p < 0.001 and ****p < 0.0001.

Several morphological differences have been described for SCVs variants including haemolytic irregularities, pigmentation, variation in colony size, slower growth rate, and differences in cell wall composition and thickness (Baddour and Christensen, 1987; Kahl, 2003; Onyango et al., 2013). In our case, both initial-WT and relapse-SCV strains were haemolytic and pigmented (Figure 2A). Regarding the colony size, the relapse-SCV isolate was 8-fold smaller than the initial isolate with a mean colony area ± standard deviation of 0.46 ± 0.1 mm^2^ vs. 3.8 ± 0.9 mm^2^ (*p* ≤ 0.01). Growth rate experiments demonstrated that the relapse-SCV isolate grows slower than the initial-WT isolate with a 2-fold increased doubling time (mean doubling time ± standard deviation of 83.7 ± 3.1 min vs. 39.5 ± 0.3 min, *p* ≤ 0.01, respectively). This difference is mainly due to the lag phase of the stable SCV strain which is 2 h longer than that of the initial, WT strain (data not shown).

Cell wall thickness of both the initial-WT and relapse-SCV strains, measured using transmission electron microscopy, revealed that the relapse-SCV strain had a significantly thicker cell wall (mean cell wall thickness ± standard deviation = 53.78 nm ± 1.4) compared to the initial-WT phenotype (29.63 nm ± 0.9, *p* ≤ 0.001) (Figure 2B). Together these results corroborate the SCV phenotype of the relapse isolate.

Experiments to highlight auxotrophy for menadione, hemin, NAD^+^ or thymidine (Kahl, 2003, 2005; Proctor et al., 2006; Sendi and Proctor, 2009) were negative. No reversion of the SCV phenotype to the normal phenotype was observed when the relapse-SCV isolate was cultured in the presence of supplemented disks.

### Genomic Comparison of Initial-WT and Stable Relapse- SCV *S. aureus* Isolates

Once the genetic backgrounds of both WT and SCV isolates were confirmed to be identical by DNA microarray analysis and *spa* typing, whole genome sequencing of both isolates was performed to assess if the SCV phenotype can be related to genetic differences. The fully closed chromosome was obtained for both the initial-WT and relapse-SCV clinical isolates (Table 1). *In silico* MLST confirmed assignation of both strains to ST30 (CC30) and confirmed the presence of an identical virulence profile (Table S1).

**Table 1:** SMRT mapping statistical summary (Post-filtering).

**Strain**	**Assembled reads**	**N50 (bp)**	**Mean read length (bp)**	**Average coverage (x)**	**Reference length (bp)**
ST20130944	74,082	21,102	15,097	331.7	2,895,375
ST20130945	74,508	20,431	14,779	332.4	2,853,507

The phenotypic resistance to penicillin and tetracycline was corroborated by the presence of (i) a conjugative *Tn916*-like transposon, in both initial-WT and stable relapse-SCV strains, which carries the *tetM* gene coding for the resistance to tetracycline; (ii) a *Tn552* transposon harboring the BlaI, BlaR, and BlaZ components of the inducible *S. aureus* β-lactamase (*bla* operon); and (iii) a smaller non-conjugative *Tn3*-like transposon which carries a second tetracycline (*tet*) resistance gene inserted at the end of an integrated plasmid which confers resistance to the heavy metals arsenic and cadmium.

#### Presence/Absence of Genes

A striking difference between the initial-WT and relapse-SCV isolates was the loss of a ~42.5 kb prophage in the genome of the stable relapse-SCV strain. Detailed analysis revealed that it was a φSa2 which shares some homology to the 46 kb φSa2 (MRSA252) (Holden et al., 2004) prophage. Like its MRSA252 counterpart, this prophage is inserted immediately downstream from a conserved lipoprotein coding gene and it does not contain any obvious virulence determinants either than a second copy of the enterotoxin A gene (*sea*), see Table S1.

#### Detection of Nucleotide Variants

To assess the integral genomic differences between the initial-WT and the relapse-SCV, we further compared the genomes of both strains by mapping the reads of the relapse-SCV isolate against the closed genome of the initial-WT isolate. This allows for the identification of single nucleotide polymorphisms (SNP) and small insertions or deletions (INDELs). Only 5 SNPs were identified, of which 2 were intergenic and 3 were intragenic, with only 1 introducing a silent mutation. The remaining 2 intragenic SNP concerned 2 distinct hypothetical proteins (Table 2). Small deletions or insertions were observed in the SCV isolate (Table 2). Small INDELs (1 bp) inducing truncated proteins, due to a frameshift change, were observed in *sasA* (coding for the surface protein A) which plays a role in the adhesion to host cells, and in *glpD* coding for an aerobic glycerol-3-phosphate dehydrogenase, which participates in biosynthetic pathways of secondary metabolites and in iron metabolism (Haley and Skaar, 2012). Deletions leading to the suppression of amino acids without the emergence of truncated protein forms were observed for an alpha-beta hydrolase (9 bp INDEL) and a putative serine protease (6 bp INDEL). More interestingly, a 9 bp (3 aa, LysGlyPro_488−490_) non-truncating deletion was found within the *rpoB* gene encoding a DNA-dependent RNA polymerase which is the target of rifampin. Multiple SNP mutations in the *rpoB* gene have been previously described and associated to an emergence of resistance to rifampicin (Wichelhaus et al., 2002; O'Neill et al., 2006; Cui et al., 2010; Gao et al., 2013). This previously unreported mutation is coherent with the resistant profile observed in the recurrent, stable SCV isolate. Moreover, a point mutation (H_481_Y) in the RpoB protein has already been reported to play a major role on virulence, persistence, bacterial gene expression, and innate immune evasion of a clinical, stable SCV isolate (Gao et al., 2013). However, this point mutation in *rpo*B co-occurs with a point mutation in the *relA* gene (Gao et al., 2010, 2013), a key player in the synthesis pathway of the intracellular signaling molecule guanosine 3′,5′ bis(diphosphate), i.e., the alarmone ppGpp involved in the stringent response (Godfrey et al., 2002; Dalebroux et al., 2010; Wolz et al., 2010). Although we did not detect any mutation in neither *rel*A gene nor in the two other small (p)ppGpp synthetases reported in *S. aureus* (*relP* and *relQ*) at the genomic level, only the point mutation in *rpoB* gene could explain the SCV phenotype of our recurrent strain.

**Table 2:** Nucleotide variations observed between initial, WT, and recurrent, stable SCV strains.

**WT**	**SCV**	**Type of mutation**	**AA change**	**Annotation**	**Phenotype**
C	T	intergenic			
G	T	intergenic			
A	G	silent	I331	acetate kinase	
A	T	nonsense	Y108STOP	hypothetical protein	
T	C	missense	K3E	hypothetical protein	
ATTAGGACC	–	deletion (3aa)		*rpoB*	rifampicin resistance
ATTCATGAG		deletion (3aa)		Alpha-beta hydrolase fold family hydrolase	
CG	–	deletion 2 nt, pseudogene		*rocD2*	
–	CATCTG	insertion (2aa)		putative bound serine protease	
A	–	deletion 1 nt, frameshift		*sasA*	
A	–	deletion 1 nt, frameshift		*glpD*	

#### Methylome

We pursued our analysis by comparing the methylome of the initial-WT and stable relapse-SCV strains to check if the SCV phenotype could be correlated with an epigenetic regulation of gene expression.

Out of a total of 1195 N^6^-Methyladenosine m6A motifs detected, 19 m6A methylated motifs were distinct between the initial-WT and the stable relapse-SCV (1.5% of differences in methylation motifs) with the initial-WT having all 19 motifs methylated while the relapse-SCV strain had none of the 19 motifs methylated. One of these motifs was located in an intergenic region and 7 methylations were found to be located in phage proteins located within the φSa2 region exclusively found in the initial-WT strain. The remainder 12 differentially methylated regions concerned genes coding for transposases (*n* = 2), exported proteins (*n* = 2), membrane proteins (*n* = 2), *yolD* family protein (*n* = 1), penicillinase biosynthesis (*n* = 1), thioredoxin, an important antioxidant protein (*n* = 1), a thiamine-phosphate synthase related to thiamine/vitamin B1 biosynthetic process (*n* = 1) and the translation factor SUA5 which is associated with double-stranded RNA binding biological pathways (*n* = 1), see Table S2.

Despite a differential pattern in the methylation status of the thiamine-phosphate synthase, no auxotrophism was observed in the stable SCV isolate. This seems to suggest that the observed methylation status does not play an essential role in the stable SCV phenotype.

Nonetheless, considering these differential methylation patterns, in particular, those affecting the response to oxidative stress, the vitamin B1 pathway and the double-stranded RNA pathways, we next thought to confirm if these methylation patterns correlated with changes in overall transcript expression of the SCV isolate.

### Transcriptomic Comparison of Initial WT and Stable SCV Relapse *S. aureus* Isolates

Messenger RNA sequencing revealed 101 genes strongly down-regulated (Log Fold Change < −2) and 61 strongly up-regulated (Log Fold Change > 2) (see Table S3).

#### Down Regulated Genes

**Figure 3:**
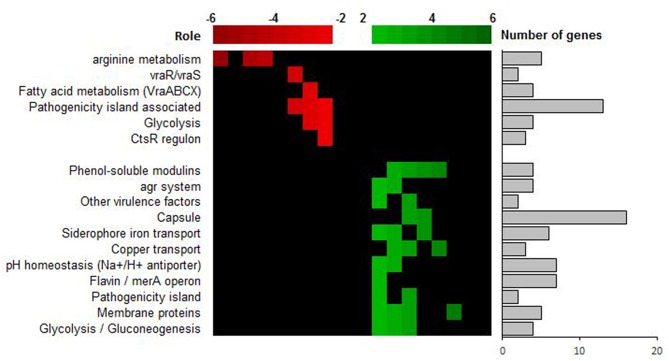
Transcriptional analysis of SCV strain ST20130945 and parental strain ST20130944. Up-regulated genes (in green) are differentially up-regulated in ST20130945 compared to the parent strain ST20130944, and the down-regulated genes (in red) are differentially down-regulated in ST20130945 compared to ST20130944. The heat map analysis highlights the role of the genes for which the differential expression was highest.

The most strongly down-regulated genes are involved in the arginine and ornithine catabolism [arginine deiminase (ADI) pathway]. This pathway permits *S. aureus* to get energy from arginine sources in glucose-free and oxygen-free conditions and leads to the production of ammonia (Makhlin et al., 2007). Other metabolic pathways such as the pyrimidine metabolism pathway, alternative carbon sources transport (phosphotransferase system PTS), carbon catabolism via the glycolytic pathway, tricarboxilic acid (TCA) pathway or the pentose phosphate pathway are also downregulated in relapse-SCV isolate compared to the initial-WT (Figure 3 and Table S3). Down-regulation of these various energy pathways may lead to ATP depletion, which have been reported in persister formation in *S. aureus* (Conlon et al., 2016). Other important down-regulated genes are involved in the cell wall regulon *vraRS* and the operon *vraABCX*, an ABC transport system under *vraRS* control. Modulation of *vraRS* expression can impact the emergence of cell-wall active antibiotic resistance in *S. aureus* (Galbusera et al., 2011;McCallum et al., 2011).

Down-regulation of energy pathway and *vraRS* and is in line with the emergence of the SCV phenotype and the switch from acute to chronic and persistence phenotype observed in clinical isolates.

#### Up-regulated Genes

The most surprising result corresponds to an upregulation of the *agr* locus and *agr*-regulated virulence genes such as delta-toxin (*hld*), PSM alpha 1 to 3, this transcriptomic profile was validated *in vitro* by an increased cytotoxicity for relapse-SCV compared to initial-WT isolate in an osteoblastic cytotoxicity test (Figure S2). Indeed, such pattern is classically associated with virulence and acute infections due to high release of staphylococcal toxins. In previous studies, we reported that intracellular release of PSM favors the cytotoxicity and that deficiency of delta-toxin is correlated with the chronicity of BJI (Rasigade et al., 2013; Valour et al., 2015). This observed up-regulation of virulence factors is in contradiction with the accepted paradigm of SCV colonies formation as *quasi*-dormant, low virulent subpopulations of bacteria.

Of note, we observed a concomitant up-regulation of genes related to the capsule formation (*cap* operon). Similar up-regulation of the *cap* operon (biofilm formation), the *agr* and alpha PSMs has already been reported in a clinical SCV strain, when compared to its WT, parental strain, and it was demonstrated that transcriptomic changes were due to a constitutively activated stringent response (Gao et al., 2013).

This high expression of phenol soluble modulins (PSM alpha and delta-hemolysin) was observed throughout the mid-exponential and (early and late) stationary phase of the stable SCV strain which further emphasizes the hypothesis of a permanently active stringent response (Figure S3). This is in contrast with the “classical” expression profile observed for the WT strain which has a high RNA III (regulatory mRNA transcript encoding the delta-toxin and part of the *agr* locus) during the exponential phase and a decreased expression on the stationary phase.

The transcriptomic pattern of the stable, SCV strain is in agreement with the 9 bp mutation in *rpo*B, the relapse-SCV phenotype and permanent stringent stress response due to a multitude of different environmental stress conditions. Moreover, it has also been demonstrated that the stringent response is activated upon internalization of *S. aureus* in human polymorphonuclear neutrophils (PMNs) with the intracellular *psm* expression contributing to survival after phagocytosis (Geiger et al., 2012).

The high expression of virulence factors by the stable-SCV strain is interesting as this strain was isolated in a context of a relapsing infection. The expression profile of virulence genes found in the stable-SCV strain is typically associated with *S. aureus* strains isolated from acute infections. Our results and the stability of the SCV phenotype observed for the relapse isolate might be associated to the “reactivation” status of a bacterial strain which has adapted to evade the host's immune response via a genetic imprinting (*rpoB* mutation) that lead to a permanent phenotype change (SCV phenotype) without compromising the strain's virulent “potential” (expression of toxins like PSM alpha, delta toxin and its major regulator, the *agr* locus). Moreover, the over expression of PSM seems pertinent as: (1) on one side they allow *S. aureus* to evade neutrophils destruction, which are the first professional phagocytic cells to be recruited into the site of bone infections; (2) on the other side, if *S. aureus* has persisted indolent via an intracellular lifestyle within osteoblast, it allows the destruction of its cellular host and the release of *S. aureus* into the extracellular bone and joint environment where it can pursue the destruction of bone tissue characteristic of BJIs. This dual potential (virulence vs. persisting, indolent status) might be achieved by a permanent activation and regulation of the stringent.

Other up-regulated genes include genes coding for ABC transporters and a multitude of genes related to the detoxification of oxidative stress associated reactive electrophilic species (RES), such as glyoxal and methylglyoxal, and/or reactive oxygen species (ROS) as well as cell wall associated lipoteichoic acid and lipid biosynthesis, glycolysis/gluconeogenesis and pH homeostasis (Figure 3 and Table S3). Again, this is interesting as (i) ABC transporters play a major role in nutrient acquisition and adaptation to rapidly changing host micro-environments (metal scavenging, amino-acids scavenging and detoxification) and (ii) reactive electrophilic species such as glyoxal and methylglyoxal are toxic chemical stressors present in the host-pathogen milieu due, in part, to the oxidative burst of activated macrophages and neutrophils and/or cellular metabolism (methylglyoxal is a highly toxic, reactive aldehyde produced as a by-product from triose-phosphate intermediates during glycolysis) (Ferguson et al., 1998; Beavers and Skaar, 2016; Imber et al., 2018). The concomitant over-expression of both ABC transporters and other detoxification genes suggests a rapid adaptation to a reactive immune system and/or changes in the pathogen's micro-environment (pH homeostasis, high concentration of toxic chemical stressors in the site of infection).

The absence of a significant up or down-regulation in the expression levels of the differentially methylated genes, in the relapse-SCV isolate, seems to suggest that the slight difference observed in the methylome of the initial-WT and relapse-SCV strains might be due to a more important role of methylation in the recognition of self and non-self DNA than in gene regulation in the evolution from acute to chronic and persistent *S. aureus* infections.

The analysis of the expression of small non-coding RNAs (ncRNA) revealed a Log Fold change (LFC) increase of 13 for the expression of the *sprG1* ncRNA, the toxin of class I toxin-antitoxin (TA) system, in the stable SCV (Table S4) The activity of type I TA toxins, most of which are small hydrophobic proteins corrupting cell envelopes, is controlled by an RNA antitoxin that inhibits translation and/or promotes degradation of toxin mRNA (Schuster and Bertram, 2016). It has been shown that *sprG1*, when over-expressed in *S. aureus* strain N315, causes cell death of *S. aureus* but also of competing bacteria and, most importantly, of erythrocytes (Pinel-Marie et al., 2014). This is interesting as the overexpression of *sprG1* is concomitant with the up regulation of high affinity iron recovery siderophores (aerobactin iron transport system, see Figure 3 and Table S3). This seems to point to a highly fine-tuned regulation of iron uptake via a dual mechanism of (1) erythrocytes' killing and (2) rescuing of newly released iron using high affinity siderophores. Iron is one of the major enzymatic co-factors in biological systems. Its acquisition is a critical determinant in staphylococcal pathogenesis and restriction to iron access is one of the host's mechanisms to resist to infection and improve its own inflammatory response (Bullen et al., 2005; Wessling-Resnick, 2010; Haley and Skaar, 2012). The ability of our relapse-SCV isolate to evade the host strategies of iron sequestration might be of major importance for its persistence within the host and evasion of the host immune system.

## Conclusion

Our stable, relapse-SCV isolate is a non-classical SCV that seems to have adopted a complex, multi-level strategy of survival and adaptation to chronicity and/or reactivation within the host. Its phenotype seems to be independent from the previously described hemin, thymidine or menadione auxotrophisms and dependent from an activated stringent response. The most obvious reason for the persistence of the strain is its *rpoB* 9bp mutation that correlates perfectly with phenotypic resistance to rifampicin and might also be responsible for a permanent activation of the stringent response, via its messenger molecule (p)ppGpp, even if we did not identify any mutation in *relA* or the other known (p)ppGpp synthetases (*relP* and *relQ*).

Thus, our clinical, stable relapse-SCV isolate seems to protect itself from the inflammatory response/oxidative stress by a plethora of pathways, including (i) RES and ROS reduction, riboflavin and NADH^+^ formation, osmoprotectants, chaperones and ABC transporters over-expression and reduced metabolism; (ii) it over expresses alpha PSM and capsule biosynthesis genes which are known to favor evasion from the immune response by impairing phagocytosis and reducing phagocytic killing; (iii) it seems to have a constitutively activated stringent response with inhibited DNA replication and a post-transcriptional inhibition via decreased ribosomal assembly and consequent inhibition of cell growth; and (iv) a down-regulation of the cell wall regulon VraRS associated to resistance to cell wall disrupting antibiotics and human cationic antimicrobial peptides (CAMPs) (Pietiäinen et al., 2009). Put together, our data strongly suggests an adaptation to chronicity, where the host immune response and antibiotic pressure are a permanent environmental stress leading to the permanent activation of the stringent response.

To further elucidate these *in silico* results more work is underway to pinpoint the modulator(s) in the stringent response pathway that are behind this stable SCV phenotype.

## Methods

### Ethics Statement

The clinical portion of this study was conducted with the approval of the French SouthEast ethics committee (no. CAL201121 for clinical isolates). In accordance with the French legislation, written informed patient consent was not required for any part of the study.

### Bacterial Strains Characterization and Growth Conditions

The two clinical *Staphylococcus aureus* isolates, collected from the acute phase [wildtype (WT) isolate ST 20130944] and the chronic phase [stable small-colony-variant (SCV) isolate ST20130945] of a BJI in the same patient, were stored at −80°C in cryotubes. Prior to all experiments, the isolates were first sub-cultivated on Columbia agar supplemented with 5% sheep blood (COS; BioMérieux) at 37°C for 24 h after thawing.

To confirm that the same bacteria was at the origin of the first, acute event and the second, relapsing event, both clinical isolates were genotyped using diagnostic DNA microarrays, identibac *S*. *aureus* Genotyping (Alere) used for this study, as well as related procedures and protocols, have been previously described in detail (Monecke et al., 2009). The assigning of isolates to CCs was determined by the comparison of hybridization profiles with those previously characterized by using multilocus sequence typing reference strains (Monecke et al., 2009). *S. aureus spa-*typing (*Staphylococcus aureus* Protein A) was also performed as previously described (Jarraud et al., 2002).

### Antimicrobial Susceptibility Testing (AST)

The AST profiles of the isolates were performed using the standard agar diffusion technique following the recommendations of the French Committee for Antimicrobial Susceptibility Testing (CA-SFM). MICs were interpreted in terms of susceptibility according to the 2016 recommendations of the EUCAST. The Minimal inhibitory concentrations for rifampin were confirmed by Etest as recommended by manufacturers.

### Colony Size Measurements

The two clinical *S. aureus* isolates (WT and SCV) were subcultivated in BHI incubated for 16 h at 36°C. After a dilution in fresh BHI of the overnight cultures to OD600 = 0.5, 100 microL of serial dilutions were inoculated in both Trypticase Soy Agar (TSA) and COS plates. After overnight incubation at 36°C, the plates were photographed, and the wild-type and SCV colonies were analyzed using an automated process in which a high-resolution picture of each plate was taken and analyzed by means of the image analysis software ImageJ (W. S. Rasband, National Institutes of Health, Bethesda, MD) (Cai et al., 2011) with a customized macro involving color thresholding, watershed algorithm, and particle analysis, in order to extract the distribution of colony areas. The WT colony area was defined as the median area of all colonies (because the median is robust to outliers, the presence of SCVs did not influence significantly this measure) while the SCVs colonies were defined as colonies with an area less than one-fifth of the median area according to the usual operational definition. Experiments were performed in triplicate.

### Auxotrophy Complementation Test

Disk diffusion assays were used to determine the auxotrophic phenotype of the stable SCV isolate for hemin, NAD, thymidine, menadione using an adaptation of the protocol previously described by Kahl et al. (1998). Briefly, colonies sub-cultivated overnight in COS were resuspended in a phosphate buffered saline (PBS, pH = 7.4) solution to 0.5 McF and 200 MicroL of bacterial suspension were spread on either Mueller-Hinton (MH) agar (BioMérieux) or Triptone/casein/Peptone (COS) (BioMérieux) media. For hemin and NAD/factor auxotrophy factor X, V and X+ V, standard disks (Sigma Aldrich, Germany) were used. For thymidine and menadione (Sigma Aldrich, Germany) auxotrophy testing disks were prepared by impregnation of 15 mL of thymidine at 100 mg/mL or menadione at 10 mg/mL (Sigma Aldrich, Germany). To determine single auxotrophism, impregnated disks were put on the MH agar surface with an inoculum of SCV isolate similar to the inoculum used for AST. auxotrophism was determined as positive if a zone of normal growth surrounding the impregnated disks was detected after 1–24 h of incubation.

### Growth Rate Determination

For the characterization of the growth dynamics of both wild type and stable SCV *S. aureus* isolates, a starter culture was prepared by inoculating colonies into BHI grown for 16 h at 36°C. Growth dynamics was performed in fresh brain-heart infusion (BHI) medium at 36°C, after dilution of overnight cultures to OD_600_ = 1.0. A thermostated microplate reader (TECAN M200 Infinite Pro) was used to follow bacterial growth by measuring OD_600_ every 15 min for 24 h. As controls, specific wells were inoculated with medium only. Experiments were performed in triplicate.

### Transmission Electron Microscopy

Both WT and stable SCV strains were prepared for TEM as described elsewhere (Gounon, 2002). and photographed by means of a JEOL 1400 microscope (Tokyo, Japan). Cell wall thickness was measured in 32 cells of each sample with nearly equatorially cut surfaces (two measures per cell) by means of the DigitalMicrograph^TM^ software (Gatan, Inc.). Cell-wall thicknesses (in nm) were compared using Student's *t*-test. The significance threshold was set at 0.05. Analyses were performed with GraphPad PRISM (version 5.0.3.477).

### Genomic DNA Preparation and Whole Genome Sequencing (WGS)

DNA extractions were performed using the QIACube R automate (Qiagen) according to the manufacturer's recommendations. DNA was eluted in molecular grade water to final concentration ≥200 ng/μL. Ion PGM sequencing—Eight 200-base-read gDNA fragment libraries were constructed using 100 ng of DNA from each sample. The libraries were prepared according to Ion XpressTM Plus gDNA Fragment Library Preparation protocol. For DNA fragmentation it was used Ion ShearTM Plus Reagents. The barcoded libraries were quantified and pooled together in even amounts. Emulsion PCR (emPCR) was carried out in the Ion OneTouchTM 2 System. The libraries were sequenced in one Ion 318TM Chip v2 using an Ion PGMTM System. For read quality assessment FastQC tool (http://www.bioinformatics.babraham.ac.uk/projects/fastqc/) was used and FASTX-Toolkit v0.0.13 (http://hannonlab.cshl.edu/fastx_toolkit/) for trimming. Single-molecule real-time (SMRT) sequencing was also performed to obtain closed genomes for both initial and recurrent strains. DNA extractions were performed using a NucleoBond^®^ PC 100 (Macherey-Nagel) according to the manufacturer's recommendations. Sequencing for *de novo* assembly was performed using PacBio RS II (Menlo Park, CA, USA). High molecular weight DNA was sheared in a Covaris g-TUBE (Covaris, Woburn, MA, USA) to obtain 20 kb fragments. After shearing, the DNA size distribution was checked using a Fragment Analyzer (Advanced Analytical Technologies, Ames, IA, USA), and 5 μg of the sheared DNA was used to prepare a SMRTbell library with PacBio SMRTbell Template Prep Kit 1 (Pacific Biosciences, Menlo Park, CA, USA) according to the manufacturer's recommendations. The resulting library was size selected using a BluePippin system (Sage Science, Inc. Beverly, MA, USA) for molecules larger than 11 kb. The recovered library was sequenced using 1 SMRT cell with P6-C4 chemistry and MagBeads with a PacBio RSII system (Pacific Biosciences, Menlo Park, CA, USA) at 240 min movie length. SMRT Mapping statistics are reported in Table 1. For *de novo* assembly, the PacBio module “RS_HGAP_Assembly.2” in SMRTpipeline version v2.3.0 was used for continuous long reads (CLR) after polishing and error correction with Quiver, as described previously (Chin et al., 2013). DNA methylation was determined using the RS_Modification_and_Motif_Analysis protocol within SMRT Portal v2.30, with a standardized *in silico* false positive error of ~1%. Only motifs with a mean modification quality value (QV) >50 and a mean coverage of >100X were validated as being modified (https://github.com/PacificBiosciences/Bioinformatics-Training/wiki/Methylome-Analysis-Technical-Note). For each strain, one polished contigs corresponding to the chromosome was obtained. Circularization of both contigs was achieved by manual comparison and removal of regions of overlaps.

### Assembly and Annotation

The gene predictions were performed using the SABIA (System for Automated Bacterial Integrated Annotation) service (Almeida et al., 2004). Further annotations were manually inspected, then checked also with SABIA.

### SNP Analysis

Ion torrent reads with at least 30 nucleotides in length were aligned with bowtie2 (Langmead and Salzberg, 2012) using “very sensitive” preset option, and one mismatch per seed region. The resultant alignment files were filtered with samtools v0.1.19 package (Li et al., 2009; Li, 2011) for mapping quality (MQ) ≥30. The SNP calling was performed with samtools mpileup and GATK (McKenna et al., 2010) tools. The PCR duplicates were next identified and marked using Picard (v. 1.51) MarkDuplicates tool (broadinstitute.github.io/picard/).The samtools mpileup parameters were minimum base quality (-Q) > 7 and skipping INDEL calling with average per-sample depth (-L) > 1,000. This was followed by filtering with bcftools SNP calling, and filtering with vcftools varFilter setting the minimum coverage (-d) of 1 and maximum (-D) of 200 reads. The GATK tool parameters were used following the Best Practice Variant Detection manual (https://www.broadinstitute.org/gatk/guide/best-practices). Three steps of calibration were performed until the number of SNPs called was stabilized. The resulting Variant Call Files (VCFs) were annotated with SnpEff tool V 4.1 (http://snpeff.sourceforge.net/). All SNPs called were manually inspected with the Integrative Genomics Viewer (IGV) tool (Robinson et al., 2011). For the manual validation of SNPs we performed the mapping of reads from the recurrent isolates into the respective initial isolate assembled genome (both SMRT and Ion Torrent reads). SNPs of “high quality” were validated using the following criteria: (1) minimum coverage ≥ 20 reads; (2) ≥75% of the reads supporting the variant call; (3) no INDELS due to homopolimeric regions 5 nts upstream or downstream to the variant call; (4) INDELS in polymeric regions were discarded (due to the limitations of the Ion Torrent technique). Manual validation of INDELS was performed using the cross match tool from phrap-consed package (http://www.phrap.org/phredphrapconsed.html). All INDELS and SNPs predicted in protein coding regions (CDS) were validated by Sanger sequencing, using primers designed especially for the amplification of the polymorphic regions.

### Detection of Presence/absence of ORFs

Was performed with the Bidirectional Best Hit (BBH) approach using BLASTp. All BLASTp searches were performed with an e-value cut off of E = 1e-05, query coverage of 90% and positives of 90%. Once the BBH pairs have been determined, we constructed the similarity clusters as follows: A cluster was defined as a set of genes in which every gene has a BBH with at least another element. Manual curation was then performed to validate the presence/absence of ORFs using the Mauve Progressive alignment algorithm (Darling et al., 2010) and the clustering analysis results.

### Detection of Mobile Genetic Elements

Distinct tools were used to characterize mobile genetic elements (MGE) such as prophages, transposons and genomic islands: PHAST (Zhou et al., 2011), Issaga (https://www-is.biotoul.fr/). Comparison with known *S. aureus* genomes showed a very high homology of both ST30 strains with the genome of strain MRSA252 (Table 2 and Figure S1), a well-characterized hospital acquired ST36 (CC30) MRSA strain representative of the highly successful epidemic EMRSA-16 clone (Holden et al., 2004). Manual curation of MGE was performed by comparison with closely related and well-annotated genome of strain MRSA252.

### RNA Preparation and Whole Transcriptome Sequencing (RNA-seq)

Overnight bacterial cultures were used to inoculate a BHI broth and incubated at 36°C with gyratory shaking at 200 rpm. Bacteria were harvested after 10 h to reach the post-exponential growth phase. Culture aliquots of 1 mL were centrifuged at 13,000 g, and the pellets were washed with 1 mL of 10 mM Tris buffer and adjusted to an optical density at 600 nm (OD_600_) of 1, corresponding to approximately 10^9^ *S. aureus* cells/mL. One mL of adjusted and washed bacterial suspension was centrifuged at 13,000 g, and the pellets were treated with lysostaphin (Sigma-Aldrich) at a final concentration of 200 mg/L. The total RNA of the pellets was then purified using the RNeasy Plus Mini Kit (Qiagen) according to the manufacturer's instructions. Removal of rRNAs was performed using the Ribo Zero kit (Illumina), following the manufacturer's instructions, and each RNA sample was suspended in 30 μL of RNA storage solution. The purity and concentration of each ribosomal depleted RNA was controlled by Bioanalyzer (Agilent) using the RNA Nano Chips, and quantity was assessed using the ND-8000 (NanoDrop Technologies). Complementary RNA libraries were prepared following the previously published protocol (Heyer et al., 2015) and quality control of the DNA colony template library was performed by cloning an aliquot into a TOPO plasmid and sequencing 8/10 clones. Samples were sequenced using a 1 ×50 bp strategy in a Hiseq2500 (Illumina, CA) sequencer. Each RNAseq library was first quality checked with the FASTQC tool (www.bioinformatics.babraham.ac.uk/projects/fastqc/) and further trimmed with Trimmomatic tool (Bolger et al., 2014) with head crop of 7 nt and minimum length of 15 nt. The resulting reads were mapped in respective *S. aureus* strain with bowtie2 tool (Langmead and Salzberg, 2012) using default parameters, excepted to one mismatch per seed region. The resulting mapping files were processed by HTSeq tool (Anders et al., 2013), in order to extract the raw counts of reads per gene. The Differential gene expression (*DGE*) analysis were carried out using the edgeR package (Robinson et al., 2010) from R programming language, as described in Anders et al. (2013). Genes were considered as differentially expressed (DEGs) when the following conditions were met: they were statistically significant as indicated by an FDR value lower than 0.05 (FDR is the False Discovery Rate, a correction of the *p*-value to account for multiple simultaneous tests) and had a change in transcript abundance of at least two (in either direction). Visual inspection of the alignment was performed using the IGV tool (Robinson et al., 2011).

### Statistical Analysis

For colony size experiments, cell wall size experiments and cytotoxicity assay, non-parametric exact Wilcoxon-Mann-Whitney tests with alpha < 0.05 were carried out to determine the significance of the results (GraphPad PRISM v 5.0.3.477).

## Data Availability Statement

The genomes of the initial WT-strain (ST20130944) and relapse-SCV (ST20130945) were submitted to the NCBI BioProject (http://www.ncbi.nlm.nih.gov/bioproject) under BioProject accessions: PRJNA497221 (strain ST20130945) and PRJNA497214 (strain ST20130944), respectively.

## Author Contributions

PS and GL performed all bioinformatic analysis and wrote the main manuscript. FV, JJ, AD, AV, and FL contributed to the main manuscript writing. ER and GL performed the RNA Seq analysis. FV prepared Figure 1. PS, ST-A, MC, and JJ performed the experiments and prepared Figures 2, 3, and Supplementary Data. All authors reviewed the manuscript.

### Conflict of Interest

The authors declare that the research was conducted in the absence of any commercial or financial relationships that could be construed as a potential conflict of interest.
