# Supplementary material for: Staphylococcus aureus Small Colony Variants (SCVs): News From a Chronic Prosthetic Joint Infection
Source: Front Cell Infect Microbiol. 2019 Oct 22;9:363. doi: 10.3389/fcimb.2019.00363 (PMC6817495; doi:10.3389/fcimb.2019.00363)
Supplement: Supplementary file 1 [file Table_1.docx]

# **Supplementary Data of ”*Staphylococcus aureus* Small Colony Variants (SCVs): news from a chronic prosthetic joint infection”**

Guilherme Loss^1^§, Patricia Martins-Simões^2,3^§, Florent Valour^3,4^, Marina Farrel Cortês^5^, Luiz Gonzaga^1^, Sophie Assant-Trouillet^6^, Jêrome Josse^3^, Alan Diot^3^, Emiliano Ricci^3^, Ana Tereza Vasconcelos^1^, Frédéric Laurent^2,3^*
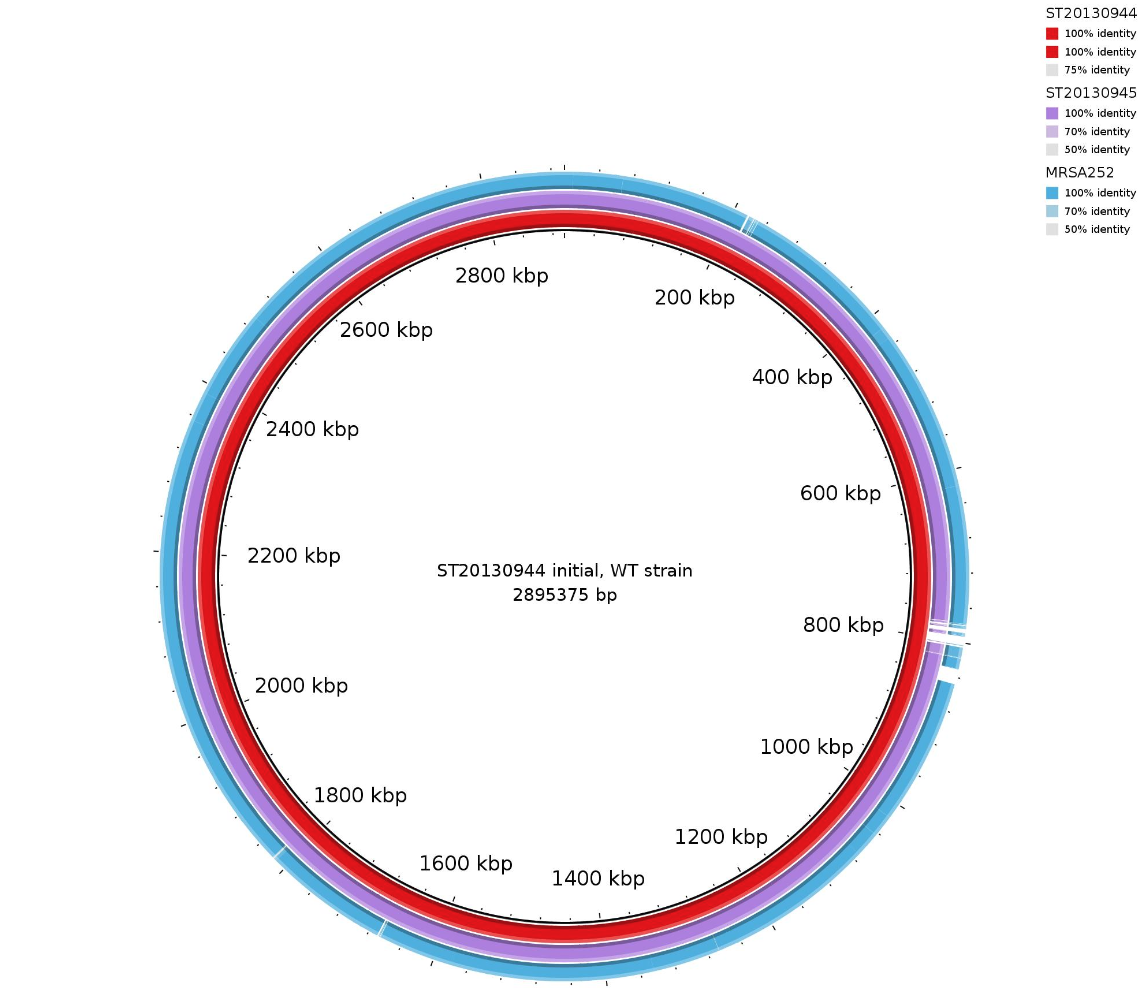


**Figure S1.** Schematic circular diagrams of the WT ST20130944, stable SCV ST20130945 and the ST36 epidemic MRSA, strain MRSA252, chromosomes.

**Validation of PSMs expression with supernatant cytotoxicity assay**

In order to confirm the production and extracellular secretion of PSM-α, bacterial supernatants from both the WT and SCV strains were put in contact with osteoblastic cell cultures, in the presence or not of serum. The supernatant obtained from the recurrent stable SCV strain was significantly more cytotoxic than the one from the initial WT strain, when cultured in BHI media (Figure S2a). However, this higher cytotoxicity was abolished in the presence of 10% of serum fetal bovine (Figure S2b), which has been previously demonstrated to inactivate PSMs (Surewaard et al., 2012 Plos Pathogen). This result strongly suggests the overexpression of PSM-α in the stable SCV strain.


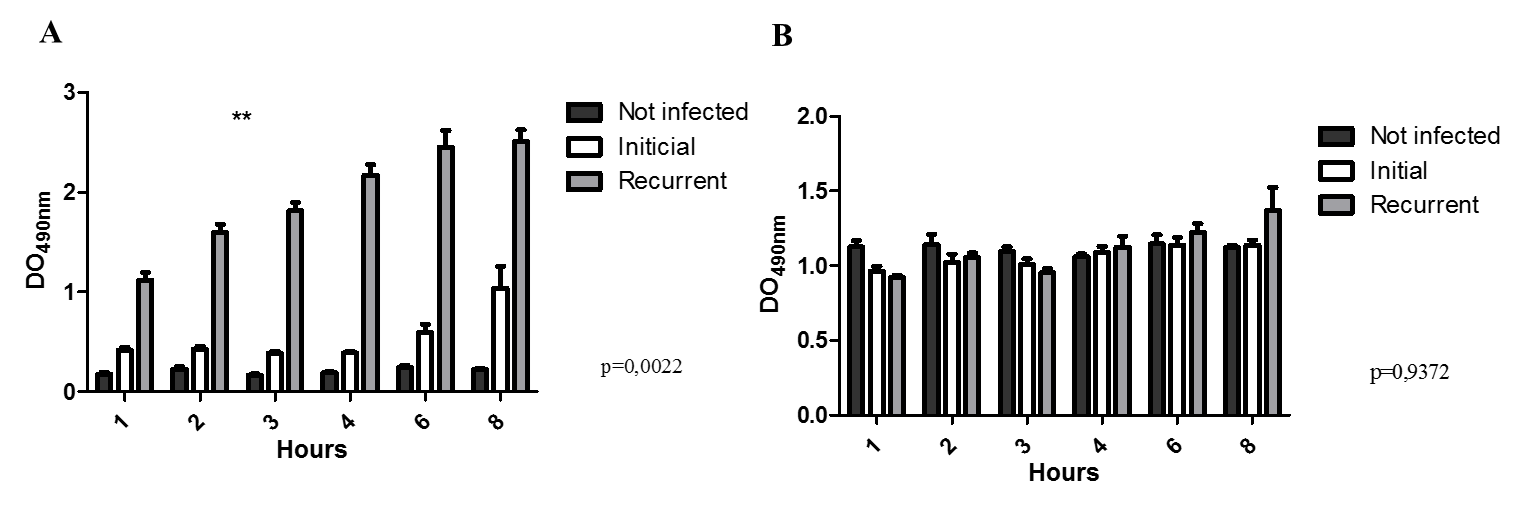


**Figure S2.** Lactate dehydrogenase (LDH) measurements in the supernatants of the osteoblast's cell culture after being in contact with bacterial supernatants of the initial and recurrent strains after 1, 2, 3, 4, 6 and 8 hours. (A) in the presence of osteoblasts' cell culture medium. (B) in the presence of osteoblasts' cell culture medium supplemented with 10% of fetal bovine serum. Statistical analysis was performed using the non-parametric exact Wilcoxon-Mann-Whitney test with alpha<0.05 to confirm the significance of the cytotoxicity differences between the WT and SCV strains

**Kinetics of PSM alpha and RNA III expression by qRT-PCR**

To validate the RNASeq experiments we decided to assess the expression kinetics of targeted genes: PSM-α operon and RNA III, a post-transcriptional regulatory RNA which encodes the delta-toxin and is part of the *agr* locus. We performed growth rate measurements at t=0, 2, 4, 6, 8, 9 and 24h from starter cultures of 45 mL of BHI broth inoculated with 1mL of overnight cultures to OD =0.1. This OD corresponds to approximately 3,7 x 10^8^ and 5.4 x 10^8^cells of the initial-WT strain and the stable-SCV strain, respectively. Based on the growth curves, we defined the time points at which each strain would be in mid-exponential, early-stationary and late stationary phases. Total RNA extractions were performed from new 45 mL BHI broth starter cultures at all 3 time points (in three independent series). We observed a high expression of both PSM-αand RNA III (δ-toxin) at all three time points for the recurrent, SCV strain. These results corroborate the RNASeq experiment but further suggest that the expression of PSM-αand *agr* associated transcripts is permanently activated in this strain (Figure S3).


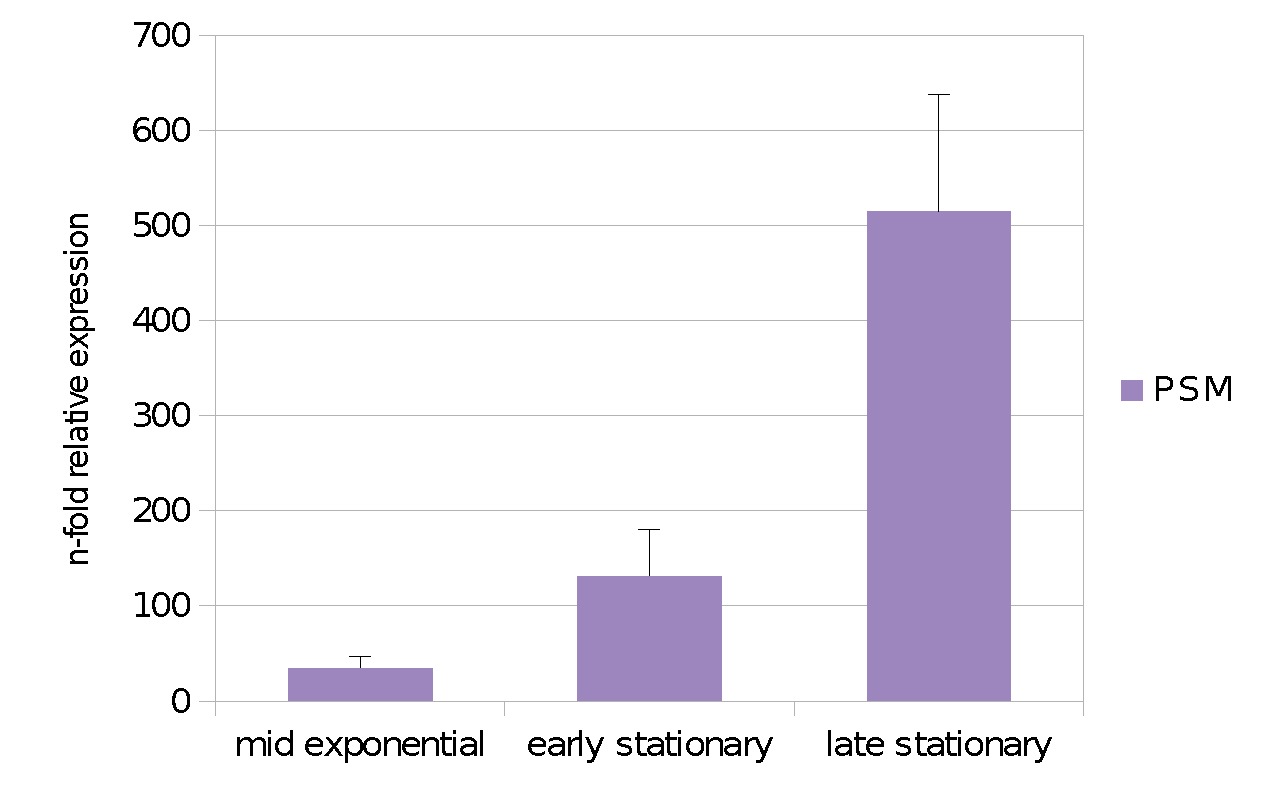

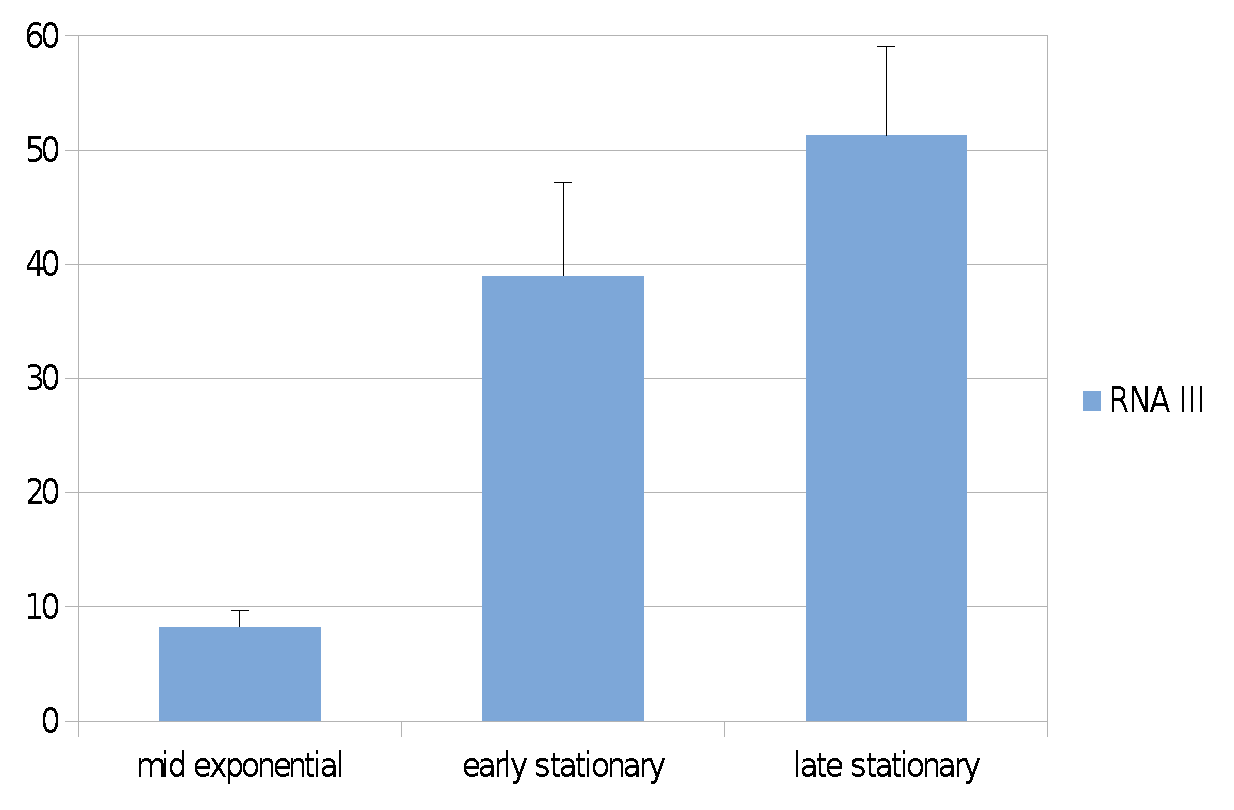


**Figure S3.** Relative transcript levels of PSM-α and RNA III (encoding the δ-toxin) were determined using quantitative reverse-transcriptase polymerase chain reaction, normalized to the internal *gyrB* standard. Results are expressed as fold change in comparison to the initial-WT strain. For each time point, experiments were performed on three independent series (biological replicates), and two RNA quantifications were done for each RNA sample (technical replicates). All values are means ± standard error of mean of 6 independent experiments.

**Materials and Methods:**

**Supernatant cytotoxicity assay.**

Cytotoxicity of both staphylococcal isolates, depending on production of exotoxins, was assessed through incubation of supernatant of bacteria with eukaryotic cells. Bacterial supernatants were obtained after centrifugation and filtration (0.22μm) of the bacterial culture in brain-heart infusion (BHI) broth under stationary phase (DO_600nm_= 2.5) for 1, 2, 3, 4, 6 and 8 hours at 37°C with 5% CO_2_**.**

Osteoblast-like cell line MG-63 was cultured in Dulbecco’s modified Eagle’s medium (DMEM) containing 2 mM**l**-glutamine and 25 mM HEPES and supplemented and 100 U/ml penicillin and streptomycin (“growth medium with antibiotics”). The MG-63 cells were seeded at 50,000 cells/well in 24-well plates and incubated at 37°C with 5% CO_2_ for 48 h in culture medium to obtain a confluence. Half of the plates had a DMEM medium further supplemented with 10% fetal bovine serum (FBS). After two washes with PBS, each well was incubated with 1ml of bacterial supernatant for 1, 2, 3, 4, 6 and 8 hours at 37°C with 5% CO_2_**.** After two washes with PBS, each well was incubated with 1ml of bacterial supernatant obtained after centrifugation and filtration (0.22μm) of the bacterial culture in brain-heart infusion (BHI) broth under stationary phase (DO_600nm_= 2.5) for 1, 2, 3, 4, 6 and 8 hours at 37°C with 5% CO_2_**.** To assess the cellular mortality, the concentration of lactate dehydrogenase (LDH) released by the damage cells was measured using the Cytotoxicity Detection Kit (Roche) in each point, according to the manufacturer's recommendations. Briefly, the supernatants of the cell culture were recovered and centrifuged at 3000g for 10 min and 100μl were applied in 96 well plates. The developing solution was prepared extemporaneously and 100μl per well was added to the supernatant, incubated for 30 min at room temperature and protected from light and then the Optical Density (OD) was measured at 490 nm. Each supernatant was tested in triplicate and the experiment was repeated three times.

The bacterial isolates were cultured in BHI à 37°C with shaking overnight.

**RNA preparation for reverse transcriptase and quantitative PCR.** For each strain, three independent bacterial cultures at an optical density at 600 nm (OD_600_) of 0.1 were used to inoculate a 45 ml BHI broth and incubated at 36°C with gyratory shaking at 200 rpm. Bacteria were harvested at mid-exponential (t=5h for the SCV strain and t=3h for the WT strain), early stationary (t=10h and t=8h for the SCV and WT strains, respectively) and late stationary (t=15h and t=13h for the SCV and WT strains, respectively) phases. Bacterial pellets of approximately 10^9^ *S. aureus* cells were washed 10 mM Tris buffer and treated with lysostaphin (Sigma-Aldrich) at a final concentration of 2,5 mg/L. The total RNA of the pellets was then purified using the RNeasy Plus Mini Kit (Qiagen) according to the manufacturer’s instructions. The RNA was quantified using a NanoDrop spectrophotometer, and 300-1000 ng of total RNA was reverse transcribed into cDNA using Reverse Transcriptase System (Promega) with random primers. Two microliters of 1/5 diluted cDNA was used as a template for the real-time quantitative polymerase chain reaction (RT-qPCR) amplification using FastStart Essential DNA Green Master kit (Roche) and the LightCycler® Nano (Roche) with specific primers shown for genes *psmα*, *RNA III* and the housekeeping gene *gyrB* (see list of priers below). Gene expression analysis was performed by using cycle threshold (∆Ct) methods using the *gyr*B gene as an internal standard. These qRT-PCR’s were performed as technical triplicates (two RNA quantification per RNA sample), on RNA obtained from three biological replicates (three independent cultures and extractions per strain per time point).

List of PCR primers used:

RNA III-F GGAAGGAGTGATTTCAATGG [1]

RNA III-R GGGATGGCTTAATAACTCATA [1]

psm alpha-F TATCAAAAGCTTAATCGAACAATTC [2]

psm alpha-R CCCCTTCAAATAAGATGTTCATATC [2]

gyrB-F GGTGGCGACTTTGATCTAGC [1]

gyrb-R TTATACAACGGTGGCTGTGC [1]

References:

[1] Song J, Lays C, Vandenesch F, et al. The expression of small regulatory RNAs in clinical samples reflects the different lifestyles of Staphylococcus aureus in colonization vs. infection. PloS One. 2012; 7(5):e37294.

[2] Dastgheyb SS, Villaruz AE, Le KY, et al. Role of Phenol-Soluble Modulins in Formation of Staphylococcus aureus Biofilms in Synovial Fluid. Camilli A, editor. Infect Immun. 2015; 83(7):2966–2975.

**Table S1.** Summary table of the general WGS features and genomic comparison between initial, WT SA isolate and recurrent, stable SCV isolate retrieved from the same patient. Abbreviations: GI = genomic islands; ф = prophages; IEC = immune evasion cluster (Schijffelen et al., BMC Genomics. 2010); °= phage inserted within the beta hemolysin encoding gene (*hlb*); inc = incomplete; Tn = transposons; IS = insertions sequences ^a^ IS1182; ^b^ IS3; ^c^ IS30; ^d^ IS6 (complete); SA = super-antigen; ent = enterotoxins; RM = Restriction-Modification system; exfol = exfoliatine.

**Table S2.** Methylated motifs present exclusively on the parental, WT isolate ST20130944**.**

**Table S3.** Significantly up and down regulated genes (false discovery rate, FDR ≤ 0.05) between the parental, WT isolate ST20130944 and the recurrent, stable SCV isolate ST20130945.

**Table S4.** Non coding RNAs with a significant up or down regulation (false discovery rate, FDR ≤ 0.05) between the parental, WT isolate ST20130944 and the recurrent, stable SCV isolate ST20130945.
